# Targeted sequencing and clinical strategies in children with autism spectrum disorder: A cohort study

**DOI:** 10.3389/fgene.2023.1083779

**Published:** 2023-02-28

**Authors:** Chunchun Hu, Yi Wang, Chunyang Li, Lianni Mei, Bingrui Zhou, Dongyun Li, Huiping Li, Qiong Xu, Xiu Xu

**Affiliations:** ^1^ Department of Child Health Care, Children’s Hospital of Fudan University, Shanghai, China; ^2^ Department of Child Health Care, Xi’an Children’s Hospital, Xi’an, China

**Keywords:** targeted sequencing, NGS, autism spectrum disorder, gene, ASXL3

## Abstract

**Objectives:** Autism spectrum disorder (ASD) is a neurodevelopmental disorder with genetic and clinical heterogeneity. Owing to the advancement of sequencing technologies, an increasing number of ASD-related genes have been reported. We designed a targeted sequencing panel (TSP) for ASD based on next-generation sequencing (NGS) to provide clinical strategies for genetic testing of ASD and its subgroups.

**Methods:** TSP comprised 568 ASD-related genes and analyzed both single nucleotide variations (SNVs) and copy number variations (CNVs). The Autism Diagnostic Observation Schedule (ADOS) and the Griffiths Mental Development Scales (GMDS) were performed with the consent of ASD parents. Additional medical information of the selected cases was recorded.

**Results:** A total of 160 ASD children were enrolled in the cohort (male to female ratio 3.6:1). The total detection yield was 51.3% for TSP (82/160), among which SNVs and CNVs accounted for 45.6% (73/160) and 8.1% (13/160), respectively, with 4 children having both SNVs and CNV variants (2.5%). The detection rate of disease-associated variants in females (71.4%) was significantly higher than that in males (45.6%, *p* = 0.007). Pathogenic and likely pathogenic variants were detected in 16.9% (27/160) of the cases. *SHANK3, KMT2A,* and *DLGAP2* were the most frequent variants among these patients. Eleven children had *de novo* SNVs, 2 of whom had *de novo ASXL3* variants with mild global developmental delay (DD) and minor dysmorphic facial features besides autistic symptoms. Seventy-one children completed both ADOS and GMDS, of whom 51 had DD/intellectual disability (ID). In this subgroup of ASD children with DD/ID, we found that children with genetic abnormalities had lower language competence than those without positive genetic findings (*p* = 0.028). There was no correlation between the severity of ASD and positive genetic findings.

**Conclusion:** Our study revealed the potential of TSP, with lower cost and more efficient genetic diagnosis. We recommended that ASD children with DD or ID, especially those with lower language competence, undergo genetic testing. More precise clinical phenotypes may help in the decision-making of patients with genetic testing.

## Introduction

Autism spectrum disorder (ASD) is a highly heterogeneous neurodevelopmental disorder characterized by social deficits and restricted, repetitive patterns of behavior and interests (Lord et al., 2020). The ASD occurrence in the United States is estimated to be approximately 1 in 44, with an overall male-to-female prevalence ratio of 3.4:12). As one of the most heritable medical conditions, ASD is associated with over a thousand risk genes (He et al., 2013), of which more than 100 genes and genomic regions meet rigorous statistical thresholds for the correlation with ASD phenotype (Satterstrom et al., 2020). Models of genetic risk for ASD tend to favor complex inheritance; nevertheless, rare inherited and *de novo* variants contribute to a substantial risk of individuals with ASD (Iossifov et al., 2014; Sanders et al., 2015). According to recently published large case‒control studies (Satterstrom et al., 2020; Fu et al., 2022; Zhou et al., 2022), the genetic contribution to ASD continues to increase. Children with a diagnosis of ASD are recommended for etiological assessments. Chromosomal microarray analysis (CMA), detecting large duplications or deletions, was used as first-tier genetic testing for children with ASD, multiple congenital anomalies (MCA) and developmental delay (DD)/intellectual disability (ID) (Miller et al., 2010), in addition to fragile X analysis and *MECP2* testing. Many physician organizations recommend next-generation sequencing (NGS) testing when CMA-based evaluation has no positive identifications. In recent years, with the remarkable maturity of technical aspects of NGS variant discovery, it has been reported that rare genetic variants can be found in up to 30% of the ASD population (Vorstman et al., 2017).

ASD is always accompanied by cooccurring conditions, such as DD/ID, language disorders, motor difficulties, attention deficit hyperactivity disorder (ADHD), and epilepsy. It is generally acknowledged that established ASD risk variants are associated with these comorbidities (Vorstman et al., 2017). Approximately 50% of children diagnosed with ASD will have ID (Shaw et al., 2021). The presence of ID and dysmorphic features are considered to account for a higher detection rate of genetic susceptibility factors contributing to ASD etiology (Tammimies et al., 2015; Husson et al., 2020). Likewise, finding genetic abnormalities may facilitate a better understanding of the pathophysiology of ASD, lead to early detection of cooccurring conditions and develop preventative guidance for children and families.

Here, we report the detection yields of the designed targeted sequencing panel (TSP) containing 568 ASD-related genes. ASD children were divided into subgroups according to clinical assessments, hoping to find the value of guidance for genetic testing and facilitate effective intervention based on pathological pathways inferred from the genetic information.

## Materials and methods

### Patients

The study included 160 patients who were diagnosed with ASD in the Department of Child Healthcare, Children’s Hospital of Fudan University, from June 2017 to March 2019 for genetic testing. The inclusion criteria for the cases were as follows: children met the criteria of ASD diagnosed by experienced pediatricians according to the Diagnostic and Statistical Manual of Mental Disorders, fifth edition (DSM-V) (American Psychiatric Association, 2013). Patients were also recommended to complete the Autism Diagnostic Observation Schedule, second edition (ADOS-2) (Lord C et al., 2012). The results of the ADOS included two subdomains: social affect (SA) and restricted and repetitive behavior (RRB). The total raw score was converted into the ADOS calibrated severity score, from 1 (none) to 10 (severe). The Griffiths Mental Development Scales (GMDS) (Griffiths, 1984; Huntley, 1996) were also performed with the consent of ASD parents. The raw scores of the 5 subscales (Locomotor (Lm), Personal and Social (P/S), Hearing and Speech (H/Sp), Eye and Hand (E/Hd), and Performance (Pf)) of the GMDS were transformed into developmental quotients (DQ). A DQ lower than 70 was considered delayed. For the selected cases, additional medical information was recorded.

### Targeted panel design

We selected 568 candidate genes in TSP as follows: genes marked from 1 to 4 in the ranking categories of the Gene-Scoring (2017) in SFARI Gene (https://gene.sfari.org/), genes predicted by the TADA model with a False discovery rate (FDR) value less than 0.3 (6, 17), and genes reported in large-scale studies (Vorstman et al., 2017; O'Roak et al., 2012; Wang et al., 2016). Genes were grouped and classified into 3 groups: “Group1-Definitive”, 66 genes ranked 1 or 2 in SFARI Gene and their FDR value < 0.05; “Group2-Probably”, 157 genes ranked 3 in SFARI Gene and their FDR value < 0.3 with more than one genetic study identified loss-of-function mutations that related to ASD; “Group3-Possible”, 345 genes ranked 4 in SFARI Gene and their FDR value < 0.3 with no loss-of-function mutation founding that had possible relationship with ASD. There were 110 genes in TSP correlated with ID (from JuniorDoc Database, http://drwang. top/) and 51 genes correlated with epilepsy (from EpilepsyGene Database, http://www.wzgenomics.cn/EpilepsyGene/).

### Targeted capture, sequencing, variants filtering and calling

Genomic DNA of participants was isolated from blood samples according to standard procedures by a QIAamp DNA Blood Midi Kit. Two hundred nanograms of genomic DNA from each individual was sheared by a Biorupter (Diagenode, Belgium) to acquire 150–200 bp fragments. The ends of the DNA fragments were repaired, and Illumina Adaptor was added (Fast Library Prep Kit, iGeneTech, Beijing, China). After the sequencing library was constructed, the whole exons were hybridized with costumed probes designed and synthesized by iGeneTech as mentioned above. Captured libraries were mixed in equal molar amounts and sequenced on an Illumina HiSeq2000 platform (Illumina, San Diego, CA) with 150 base paired‐end reads. The average on-target sequencing rate was 98.7%, and the target bases covered at >=20X and >=10X were 97.7% and 97.6%, respectively. Raw reads were filtered to remove low-quality reads by using FastQC. Then, clean reads were mapped to the reference genome GRCh37/Hg19 by using BWA. After removing duplications, SNVs and InDels were called and annotated by using GATK. The variants were interpreted according to ACMG guidelines (Richards, et al. Genetics in Medicine (2015) 17, 405) and patient phenotypes and were classified as pathogenic (P), likely pathogenic (LP), variants of unknown significance (VUS), likely benign (LB) or benign (B). A CNV kit was used to call the large copy number variations (CNVs), and the default parameters were used. To identify CNVs, part of the sequencing library was sequenced directly, and each sample yielded 1G raw data. CNVs were called by using CNVseq, and the controls were the healthy parents. For the diagnostic SNVs of patients and parents, Sanger sequencing was used for variant confirmation. For diagnostic CNVs, qPCR/MLPA was performed.

### Statistical analysis

Conventional descriptive statistical methods were used for presenting characteristics of the study cohort. We used unpaired t-test or Mann–Whitney *U* test depending on normality for the comparisons of ADOS scores and DQs of the GMDS in subgroups with positive and negative genetic findings in DD/ID subgroup. For categorical variables, the sex and subgroup differences of detection yields were compared by chi-squared tests. Data were presented as the mean ± standard deviation (SD) or medians and interquartile ranges (IQR) for continuous variables according to whether the data were normally distributed. Data were presented as percentages for categorical variables. Data analysis was performed using SPSS 22.0 (IBM, Armonk, NY, United States).

## Results

### Cohort description and detection yields

A total of 160 children who were diagnosed with ASD were included in the cohort (125 males and 35 females). The mean age of the patients was 3.24 ± 1.27 years. Ninety-four children completed the ADOS-2, and 75 children were assessed using the GMDS, with 71 children having both ADOS-2 and GMDS assessments (Table 1).

**TABLE 1:** Clinical characteristics and the results of ADOS/GMDS of ASD patients in the cohort.

Variables	Number (%)	Summary
Age (months)		
Males^a^	125 (78.12%)	34.00 (27.00, 50.00)
Females^a^	35 (21.88%)	34.00 (27.00, 41.00)
ADOS-2	94 (58.75%)	
Score of SA^a^		17.00 (14.00, 19.00)
Score of RRB^a^		2.00 (1.00, 3.00)
Total Score^a^		7.00 (6.00, 8.00)
Calibrated Severity Score^a^		19.00 (16.00, 21.00)
GMDS	75 (46.88%)	
DQ of Lm^b^		70.76 ± 16.88
DQ of P/S^a^		52.00 (44.50, 68.75)
DQ of H/Sp^a^		38.00 (30.00, 56.50)
DQ of E/Hd^b^		59.80 ± 22.90
DQ of Pf^a^		63.00 (52.00, 84.50)
Total DQ^b^		60.44 ± 18.36

**SD**, standard deviation; **ADOS**, autism diagnostic observation schedule; **SA,** social affect; **RRB,** restricted repetitive patterns of behavior; **DQ,** developmental quotient; **GMDS,** griffiths mental development scales; **Lm,** Locomotor; **P/S,** personal and social; **H/Sp,** Hearing and Speech; **E/Hd,** Eye and Hand; **Pf**, Performance.

^a^ Median (IQR).

^b^ Means ± SD.

The overall detection yield of TSP was 51.3% (82/160) for analyzing both SNVs and CNVs, of which 57 were male and 25 were female. Although the number of males was higher than that of females, the detection rate of disease-associated variants in females (71.4%) was significantly higher than that in males (45.6%, *χ2* = 7.30, *p* = 0.007). The “pathogenic/likely pathogenic (P/LP)” rate was 16.9% (27/160), including 16 males and 11 females. For SNVs, 73 children had positive results (24 females), accounting for a detection rate of 45.6%, of which “P” variants were found in 4.4% of cases (7/160), “LP” in 8.1% of cases (13/160), and “VUS” in 33.1% of cases (53/160). We identified a total of 90 SNVs, of which 74 were missense mutations, 5 were frameshift mutations, and 11 were splicing mutations. The most common variants were *DLGAP2, SHANK3,* and *KMT2A*, which were present in 3 probands for each variant. Of 73 patients with SNVs, 68 patients underwent parental testing (both father and mother), and 3 patients had variant confirmation only by mother. We found 11 cases carried *de novo* variants, including *ASXL3, KMT2A,* and *MECP2*, which accounted for 16.2% (11/68) of the analyzed trios. Of the other 74 variants, 42 were of maternal origin, and 30 were of paternal origin, with 2 variants of non-maternal origin (lack of paternal samples) (Table 2). CNVs were found in 13 patients (3 females), which accounted for 8.1% (13/160), whereas 4 children had dual SNV and CNV. The percentage of pathogenic CNVs was 15.4% (2/13), “LP” was 46.2% (6/13) and “VUS” was 38.5% (5/13). A total of 23.1% (3/13) were duplication variants, and 76.9% (10/13) were heterozygous deletions. 17p11.2 had the greatest number of reportable CNVs, accounting for 46.2% (6/13) (Table 3).

**TABLE 2:** SNVs identified from TSP.

patient	Sex	Gene	Variant segregation	Position	Mutation	Inheritance pattern	Zygosity
P							
1	F	*MECP2*	*de novo*	ChrX:153296806	NM_004992.3:exon4:c.473C>T:p.T158M	XL	Het
2	F	*MECP2*	*de novo*	ChrX:153296362	NM_004992.3:exon4:c.917G>A:p.R306H	XL	Het
3	F	*PTEN* P)	maternal	Chr10:89720857	NM_000314.7:exon8:c.1008C>G:p.Y336X	AD	Het
		*CUL9* (VUS)	—	Chr6:43154146	NM_015089.4:exon4:c.1204G>A:p.D402N	--	Het
		*NRXN2* (VUS)	—	Chr11:64434827	NM_138732.3:exon8:c.1600C>A:p.L534I	--	Het
4	M	*ASXL3* P)	*de novo*	Chr18:31324172	NM_030632.3:exon12:c.4360C>T:p.Q1454X	AD	Het
		*KMT2A* (VUS)	paternal	Chr11:118348825	NM_001197104.1:exon5:c.3478G>A:p.G1160S	AD	Het
5	M	*ASXL3*	*de novo*	Chr18:31324212	NM_030632:exon12:c.4400_4403dup:p.P1470Nfs*4	AD	Het
6	F	*SHANK3*	*de novo*	Chr22:51160241	NM_001372044.2:exon24:c.4209del:p.S1404fs	AD	Het
7	F	*TRIP12*	*de novo*	Chr2:230744795	NM_004238.3:exon2:c.1A>G:p.M1V	AD	Het
LP							
8	F	*CHD4*	not maternal	Chr12:6697033	NM_001273.5:exon24:c.3548G>A:p.R1183H	AD	Mosaic (25%)
9*	F	*CHD1* (LP)	paternal	Chr5:98228317	NM_001270.2:exon14:c.2092G>A:p.V698I	AD	Het
		*CTNND2* (VUS)	maternal	Chr5:11411688	NM_001332.4:exon5:c.399A>C:p.E133D	--	Het
		*DLGAP2* (VUS)	maternal	Chr8:1616837	NM_001346810.2:exon9:c.2153G>A:p.G718E	--	Het
10	F	*DDX53*	maternal	ChrX:23018700	NM_182699.4:exon1:c.530_549del:p.N177fs	--	Het
11	M	*TMLHE*	maternal	ChrX:154741452	NM_018196.4:exon5:c.640G>T:p.E214X	XL	Hem
12	M	*KIRREL3*	maternal	Chr11:126396583	NM_032531.4:intron2:c.134-1G>A	--	Het
13	M	*SCN8A* (LP)	paternal	Chr12: 52167979	NM_014191.4: exon20: c.3652G>T: p. E1218X	AD	Het
		*FOXP2* (VUS)	paternal	Chr7:114284820	NM_014491.4: exon8: c.1070G>A: p.C357Y	AD	Het
14	M	*KIRREL3*	paternal	Chr11:126310421	NM_032531.4:exon11:c.1276C>T:p.Q426X		Het
15	M	*KMT2A*	*de novo*	Chr11:118392670	NM_001197104.1:exon36:c.11702A>C:p.H3901P	AD	Het
16	M	*NLGN4X* (LP)	*de novo*	ChrX:5821448	NM_020742.3:exon5:c.1271A>C:p.Y424S	Mu/XL	Het
		*MAST1* (VUS)	maternal	Chr19:12949464	NM_014975.3: exon1: c.79_80del: p. K27fs	AD	Het
17	M	*KMT2A*	*de novo*	Chr11:118392670	NM_001197104.1:exon36:c.11702A>C:p.H3901P	AD	Het
18	M	*KMT2C*	*de novo*	Chr7:151927409	NM_170606.3:intron16:c.2770-4dup	AD	Het
19	M	*SND1*	not maternal	Chr7:127724827	NM_014390.4:exon19:c.2168del:p.P723fs	--	Het
20	F	*GABRB3* (LP)	*de novo*	Chr15:26874148	NM_001191321.3:exon1:c.3G>A:p.M1I	AD	Het
		*SLC4A8* (VUS)	maternal	Chr12:51847363	NM_001039960.3: exon5: c.454C>T: p.R152C	--	Het
VUS							
21*	M	*SCN3A*	paternal	Chr2:165946956	NM_006922.4:exon28:c.5707T>A:p.S1903T	AD	Het
22	F	*CNTNAP5*	maternal	Chr2:125262049	NM_130773.4:exon8:c.1240G>C:p.G414R	--	Het
23	F	*CDH8*	maternal	Chr16:61854979	NM_001796.5:exon6:c.874G>A:p.G292S	--	Het
24	F	*SLC6A4*	paternal	Chr17:28536176	NM_001045.6:exon12:c.1534G>C:p.V512L	AD	Het
25	F	*CNTNAP5*	paternal	Chr2:125669071	NM_130773.4:exon23:c.3680A>T:p.E1227V	--	Het
26	F	*USP15*	—	Chr12:62696710	NM_001252078.2:exon3:c.348 + 9T>C	--	Het
		*CACNA1G*	—	Chr17:48685339	NM_018896.5:exon25:c.4664G>A:p.R1555Q	AD	Het
27	F	*RERE*	maternal	Chr1:8418649	NM_012102.4:exon21:c.3946G>A:p.E1316K	AD	Het
28	M	*MYH10*	maternal	Chr17:8452042	NM_001256012.2:exon10:c.983C>T:p.P328L	--	Het
29	F	*RELN*	paternal	Chr7:103629592	NM_005045.4:exon1:c.212G>T:p.G71V	AD/AR	Het
30	F	*POLRMT*	paternal	Chr19:619653	NM_005035.4:exon13:c.2999G>C:p.G1000A	--	Het
		*MYO9B*	paternal	Chr19:17265185	NM_001130065.2:exon6:c.1159G>A:p.A387T	--	Het
31	F	*ADCY9*	—	Chr16:4029173	NM_001116.4:exon8:c.2623C>T:p.L875F	--	Het
32	F	*PHF10*	paternal	Chr6:170114835	NM_018288.4:exon7:c.797A>G:p.Y266C	--	Het
33	F	*AFF2*	paternal	ChrX:148037716	NM_002025.4:exon11:c.2141A>C:p.D714A	XL	Het
34	F	*NR3C2*	maternal	Chr4:149035254	NM_000901.5:intron8:c.2799 + 1G>A	AD	Het
		*SLC6A8*	paternal	ChrX:152955824	NM_005629.4:exon2:c.263–6C>T	XL	Het
35	M	*ARVCF*	paternal	Chr22:19965558	NM_001670.3:exon8:c.1621C>T:p.R541W	--	Het
		*DAAM2*	maternal	Chr6:39832252	NM_001201427.2:exon4:c.302A>G:p.Y101C	--	Het
36	M	*NINL*	maternal	Chr20:25450631	NM_025176.6:exon18:c.3349C>T:p.Q1117X	--	Het
37	M	*DLGAP2*	maternal	Chr8:1513907	NM_001346810.2:exon6:c.1289G>T:p.C430F	--	Het
38	M	*TRPM5*	maternal	Chr11:2436135	NM_014555.3:exon10:c.1620 + 2T>C	--	Het
39	M	*KMT2C*	maternal	Chr7:151851399	NM_170606.3:exon47:c.12092C>G:p.P4031R	AD	Het
40	M	*DDX3X*	maternal	ChrX:41196733	NM_001193416.3:intron2:c.103 + 15T>C	XL	Hem
41	M	*NSD1*	maternal	Chr5:176707762	NM_022455.4:exon18:c.5819A>C:p.Q1940P	AD	Het
42	M	*PRODH*	maternal	Chr22:18900719	NM_016335.5:exon15:c.1772G>C:p.R591P	AD/AR	Het
		*PRODH*	paternal	Chr22:18908862	NM_016335.5:exon9:c.1004A>G:p.N335S	AD/AR	Het
43	M	*AGO1*	paternal	Chr1:36358887	NM_012199.5:intron4:c.512 + 8G>A	--	Het
44	M	*TNRC6B*	paternal	Chr22:40662956	NM_001162501.2:exon5:c.2722A>T:p.N908Y	AD	Het
45	M	*SHANK3*	maternal	Chr22:51137156	NM_033517.1:exon12:c.1495G>A:p.V499M	AD	Het
46	M	*CHD7*	maternal	Chr8:61734352	NM_017780.4:exon10:c.2701G>A:p.V901M	AD	Het
47	M	*NRXN3*	paternal	Chr14:79432509	NM_004796.6:exon9:c.1418T>A:p.I473N	--	Het
48	M	*CHD8*	maternal	Chr14:21871792	NM_001170629.2:exon17:c.3338G>A:p.R1113H	AD	Het
49	M	*PTPRM*	maternal	Chr18:7926611	NM_001105244.1:exon5:c.593C>G:p.A198G	--	Het
50	M	*NRXN2*	maternal	Chr11:64453401	NM_138732.3:exon5:c.797C>T:p.A266V	--	Het
51	M	*CACNA1H*	maternal	Chr16:1257363	NM_021098.3:exon14:c.2996T>C:p.M999T	AD	Het
52	M	*SOX5*	maternal	Chr12:23908608	NM_006940.6:exon4:c.532C>T:p.L178F	AD	Het
53	M	*SHANK3*	maternal	Chr22:51159239	NM_033517.1:exon21:c.2936G>T:p.R979L	AD	Het
54	M	*NRXN1*	maternal	Chr2:50149270	NM_001135659.2:exon24:c.4456C>A:p.L1486I	AR	Het
		*NRXN1*	paternal	Chr2:50280420	NM_001135659.2:exon22:c.4237C>T:p.P1413S	AR	Het
		*SCN8A*	paternal	Chr12:52159495	NM_014191.4:exon16:c.2585A>G:p.N862S	AD	Het
55	M	*SCN2A*	paternal	Chr2:166210993	NM_021007.3:exon17:c.3211G>A:p.G1071R	AD	Het
56	M	*SOS1*	paternal	Chr2:39213067	NM_005633.3:exon23:c.3900A>T:p.Q1300H	AD	Het
57	M	*DDX3X*	maternal	ChrX:41205889	NM_001193416.3:intron14:c.1615 + 14T>C	XL	Hem
58*	M	*ASH1L*	paternal	Chr1:155491209	NM_018489.3:exon2:c.102G>C:p.K34N	AD	Het
59	M	*EP400*	maternal	Chr12:132446252	NM_015409.5:exon2:c.1088A>C:p.Q363P	AD	Het
60	M	*EPB41L3*	maternal	Chr18:5478269	NM_012307.4:exon3:c.352G>C:p.D118H	--	Het
61	M	*SIN3A*	maternal	Chr15:75688840	NM_001145358.2:intron13:c.1855–3C>G	AD	Het
62	M	*RELN*	paternal	Chr7:103143520	NM_005045.4:exon52:c.8432T>C:p.F2811S	AD/AR	Het
63*	M	*DPP6*	maternal	Chr7:154561214	NM_001936.5:exon9:c.785C>G:p.P262R	AD	Het
		*LZTR1*	paternal	Chr22:21342407	NM_006767.4:exon5:c.509G>A:p.R170Q	AD/AR	Het
64	M	*SCN3A*	maternal	Chr2:166011041	NM_006922.4:exon11:c.1301C>A:p.T434N	AD	Het
65	M	*SEMA5A*	paternal	Chr5:9108299	NM_003966.3:exon16:c.2026C>T:p.R676C	--	Het
66	M	*SLC6A8*	maternal	ChrX:152959811	NM_005629.4:exon10:c.1405G>C:p.V469L	XL	Hem
67	M	*SKI*	paternal	Chr1:2237536	NM_003036.4:exon6:c.1845G>T:p.E615D	AD	Het
68	M	*SCN1A*	maternal	Chr2:166912967	NM_001165963.3:exon6:c.427G>A:p.V143M	AD	Het
69	M	*SOS1*	paternal	Chr2:39285930	NM_005633.3:exon3:c.229A>T:p.S77C	AD	Het
70	M	*DLGAP2*	maternal	Chr8:1496851	NM_001346810.2:exon5:c.232C>T:p.P78S	--	Het
71	F	*CHD8*	maternal	Chr14:21894278	NM_001170629.2:exon5:c.1716 + 9A>T	AD	Het
72	M	*LRRC1*	maternal	Chr6:53767467	NM_018214.5:exon9:c.828T>A:p.N276K	--	Het
73	F	*EP400*	paternal	Chr12:132446354	NM_015409.5:exon2:c.1190A>G:p.Q397R	--	Het
		*SRCAP*	maternal	Chr16:30735196	NM_006662.3:exon25:c.4451T>A:p.V1484D	AD	Het

**SNV,** single nucleotide variations; **P,** pathogenic**; LP,** likely pathogenic**; VUS,** variants of unknown significance; **M**, male; **F**, female; **AD**, autosomal dominant; **AR**, autosomal recessive; **XL**, X-linked; **Het**, heterozygous; **Hem**, Hemizygous. *, patients with both SNVs, and CNVs.

**TABLE 3:** CNVs in ASD patients from TSP.

patient	Sex	Chromosome location	Position	Size	Deletion/duplication
P					
1	F	15q13.3	chr15:29346088–32460659	3.11 Mb	deletion
2	M	22q13.3	chr22:49895953–51135096	1.24 Mb	deletion
LP					
3	M	17q11.2	chr17:29483001–29687721	204.72 Kb	deletion
4	M	17q11.2	chr17:29483001–29687721	204.72 Kb	deletion
5	M	Xp11.2	chrX:47435744–47473973	38.23 Kb	deletion
6	F	6q27	chr6:164539952–170155049	5.62 Mb	deletion
7	M	17q11.2	chr17:29483001–29657516	174.52 Kb	deletion
8*	F	17q11.2	chr17:29483001–29665823	182.82 Kb	deletion
VUS					
9*	M	8q22.2	chr8:100844597–100887894	43.3 Kb	duplication
10	M	7q36.1	chr7:151833917–151960215	126.3 Kb	duplication
11*	M	22q11.2	chr22:19168244–19263353	95.11 Kb	duplication
12	M	17q11.2	chr17:29483001–29664600	181.6 Kb	deletion
13*	M	17q11.2	chr17:29483001–29687721	204.72 Kb	deletion

*: patients with both SNV and CNV (patient 8, 9, 11, 13 were patient 9,21, 58 and 63 in Table 2); **CNV,** copy number variations; **P,** pathogenic**; LP,** likely pathogenic**; VUS,** variants of unknown significance**; M**, Male; **F**, Female.

### Comparison of language competence in children with and without genetic abnormalities in DD subgroup

Among 71 children, the average DQ of the GMDS was 61.26 ± 17.43, and the calibrated severity score of the ADOS-2 was 7.14 ± 1.45. The detection yield of these 71 children was 54.9% (39/71), with P/LP variants reached 19.7% (14/71). The detection rate of this subgroup was not statistically different from that of general ASD cohort (*χ2* = 0.77, *p* = 0.774). There were 51 patients (71.8%, 51/71) with a total DQ under 70. In this subgroup of ASD children combined DD, 30 patients had positive genetic variants (58.8%, 30/51), with P/LP rate reaching 21.6% (11/51). Children with genetic abnormalities had lower language competence than children without positive genetic findings (*Z* = -2.20, *p* = 0.028). There were no correlations between ASD symptoms and the detection of genetic abnormalities in this DD subgroup (Table 4).

**TABLE 4:** Comparison of ASD symptoms and developmental scores between children with positive and negative genetic variants in DD/ID subgroup.

	Genetic findings		*χ2*/t/Z	*p*-value
	Positive (n = 30)	Negative (n = 21)		
Sex^a^			2.71	0.100
Male (%)	22 (43.1%)	20 (39.2%)		
Female (%)	8 (15.7%)	1 (2.0%)		
Age^b^	41.50 (27.00, 53.00)	40.00 (28.00, 59.00)	0.15	0.878
ADOS				
Score of SA^b^	17.50 (16.00, 19.00)	17.00 (13.00, 18.00)	1.40	0.162
Score of RRB^b^	2.50 (1.00, 3.00)	2.00 (1.00, 4.00)	0.15	0.878
Total score^b^	7.00 (6.25, 8.00)	7.00 (6.00, 8.00)	0.81	0.419
Calibrated severity score^b^	20.00 (17.00, 21.00)	18.00 (15.00, 22.00)	0.88	0.377
GMDS				
DQ of Lm^c^	63.83 ± 13.32	69.10 ± 8.64	1.59	0.118
DQ of P/S^c^	47.27 ± 14.52	54.43 ± 13.76	1.77	0.083
DQ of H/Sp^b^	30.50 (23.00, 40.25)	35.00 (32.00, 48.00)	2.20	**0.028** **^d^**
DQ of E/Hd^c^	49.33 ± 16.39	54.95 ± 10.24	1.39	0.139
DQ of Pf^b^	61.00 (40.25, 66.75)	56.00 (49.00, 60.00)	0.55	0.585
Total DQ^c^	51.33 ± 13.44	55.59 ± 7.52	1.31	0.155

**DD/ID,** developmental delay/intellectual disability; **ADOS**, autism diagnostic observation schedule; **SA, s**ocial affect; **RRB,** restricted repetitive patterns of behavior; **GMDS,** griffiths mental development scales; **Lm,** Locomotor; **P/S,** personal and social; **H/Sp,** Hearing and Speech; **E/Hd,** Eye and Hand; **Pf**, Performance; **DQ**, developmental quotient.

^a^ Categorical variables were compared with a Chi-square test.

^b^ The differences of skewed distributed continuous variables (shown as medians and interquartile ranges) are tested by the Mann-Whitney U tests.

^c^ The differences of normally distributed continuous variables (shown as means ± SD) are tested by unpaired t tests.

^d^ *p* < 0.05.

### De novo variants of *ASXL3* in two patients

**FIGURE 1:**
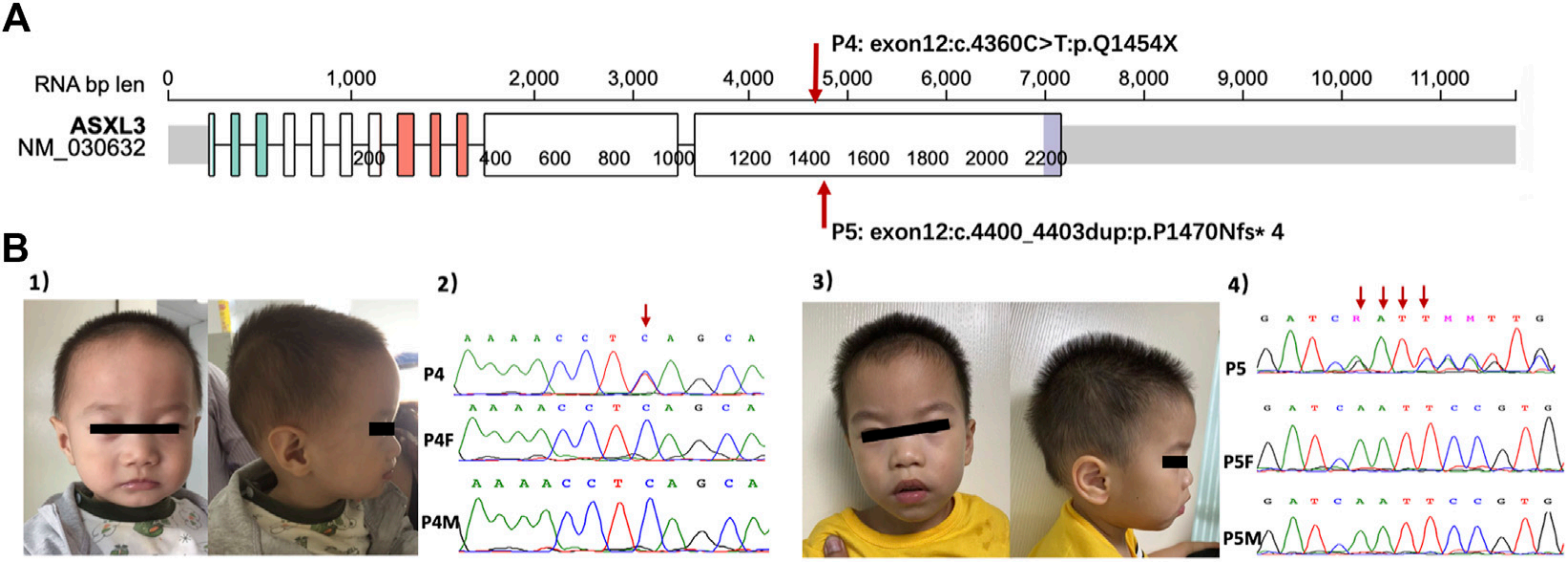
**(A)** The location of *ASXL3* variants in patients based on Protein Paint (https://proteinpaint.stjude.org, access in September 29th). The numbers of patients correspond with Table 2. **(B) (1)** The facial features of P4. **(2)** Sanger sequence of P4 and his parents with *ASXL3* genetic locus. The red arrow showed the mutation stie of P4. **(3)** The facial features of P5. **(4)** Sanger sequence of P5 and his parents with *ASXL3* genetic locus. The red arrows showed the duplication sties of P5. P4, patient 4; P4F, father of patient 4; P4M, mother of patient 4; P5, patient 5; P5F, father of patient 5; P5M, mother of patient 5.

Patient 4 and Patient 5 had *de novo* variants of *ASXL3* (Figure 1). Patient 4 was referred to our clinic at 19 months for delayed development. He had poor eye contact as well as response to names. Repetitive behaviors included stamping and shanking head/hands. Tracing the developmental milestones, the patient was unable to crawl and pull up to stand at that time and he learned to sit without support until the age of 10 months. He could only make repeated single-syllable sounds. His birthweight was normal, but feeding seemed very difficult in the early stage, resulting in poor postnatal growth (2 SD below the mean). Physical examination showed that he had a prominent forehead, wildly spaced eyes, strabismus and malformation of external auditory canals. When he had reexamination at 6 years old, he still had language delay. An oral examination revealed that he had dental overcrowding. The Wechsler Preschool and Primary Scale of Intelligence (WPPSI) (Wechsler, 2012) showed that his intelligence quotient (IQ) was 69. Patient 5 was a 2.7-year-old boy. He displayed repetitive behaviors such as throwing and biting objects, turning the wheels and sometimes squinting. He had obvious delayed speech and language development because he was non-verbal at the time of referral. Feeding difficulty also happened to him. Walking independently was at the age of 20 months. His total DQ of the GMDS was 55.2 (the DQs of all the subscales were less than 70). Facial dysmorphism was prominent forehead but there was no obvious deformity in other parts. He had febrile convulsions twice, while electroencephalogram (EEG) and magnetic resonance imaging (MRI) were normal. Two patients shared the common characteristics of *ASXL3* variants, but they had only mild ID/DD, which was noteworthy.

## Discussion

In this study, we investigated the detection yields and novel variants through TSP of 568 ASD-associated genes in an ASD cohort. The detection yield was 51.3% in TSP, with the rate of “P/LP” reaching 16.9%. With the falling costs of sequencing, more patients with neurodevelopmental disorders are allowed to receive genetic testing whose positive results give them better access to new treatments. CMA was considered the appropriate initial test for the etiologic evaluation of ASD children (Hyman et al., 2020). There is increasing evidence that NGS, whole-exome sequencing (WES) and whole-genome sequencing (WGS) offer diagnostic advantages over CMA (22). Sirvstava et al.‘s review (Srivastava et al., 2019) revealed a yield in the range of 30%–40% for exome sequencing, which exceeds the 10%–20% yield for CMA. Feliciano et al. (2019) conducted WES in 457 ASD families with genetic identification in 15.2% multiplex families and 10.1% simplex families. According to Ghralaigh’s study (Ni Ghralaigh et al., 2020), the diagnostic yield in ASD was 31% using WES and 42.4% using WGS, but the cost estimates were €79.33 and €1239.5 for choosing different technologies. For panel sequencing, a meta-analysis by Stefanski et al. (2021) showed that the identification of genetic defects accounted for 22.6%, compared to 27.2% for WES. Speak frankly, WES and WGS have higher diagnostic yields of ASD than panel sequencing; however, the benefits do not outweigh their drawbacks. WES and WGS offer higher costs than panel sequencing; on the other hand, due to the larger amount of data, more time is required for analysis and processing. Therefore, an affordable sequencing panel that can capture relevant genes may be a good compromise. It can not only achieve molecular diagnosis and detection efficiency in less cost and time but also avoid the waste of resources. The most important factor in ASD families’ decision about genetic testing is cost. Sequencing panel is still the most cost-effective choice. Ghralaigh et al.‘s report (Ni Ghralaigh et al., 2022) demonstrated 0.22%–10.02% diagnostic yields of gene panels to derive the conclusion that gene panels marketed for use in ASD are currently of limited clinical utility. However, gene selection and numbers for inclusion of gene panels are the key factors for results. A well-defined/comprehensive gene set is required in gene panels. We selected genes with the most promising diagnostic purpose of ASD. The most frequent variants in our cohort were *SHANK3, KMT2A,* and *DLGAP2*, which was a slightly different from the previous ASD cohort studies (Satterstrom et al., 2020; Fu et al., 2022). ASD frequent genes like *SCN2A, CHD8, PTEN* and so on were identified in our cohort whereas we did not find *SYNGAP1, ADNP* variants according to our sample size. For 72 genes associated with ASD at FDR value <=0.001 in Fu et al.’ s study (Fu et al., 2022), we have 51genes overlapped in our panel. Of identified 102 risk gene in Satterstrom’s study (Satterstrom et al., 2020), 66 of them overlapped with our TSP. Our designed TSP including most of the ASD frequent genes and whose detection yield reached 51.3%, is specialized for ASD patients, and can be considered a success for panel sequencing and potential for the clinical utility of ASD.

Although the reported prevalence sex ratio is four times higher in males than in females (Brugha et al., 2016), we observed that the detection rate of genetic variants was 1.5 times higher in females than males. Sex differences were also observed in other genetic studies (De Rubeis et al., 2014; Satterstrom et al., 2020). The possible reasons were that cognitive defects and autistic traits in females are less severe than those in males. Conversely, females may need clearer autistic characteristics and comorbid DD/ID to receive a diagnosis of ASD. It is believed that sex differences are consistent with the female protective effect model, which assumes that women need an increased genetic load to reach the threshold for ASD diagnosis (Werling, 2016). Thus, more prominent phenotypes demonstrate a higher risk for genetic variants in females than males with ASD.

Language plays a major part in the outcomes of ASD. ASD children whose language is impaired, could have a large impact on the social interaction and general wellbeing of individuals (Nudel et al., 2021). Improvements in the language of ASD children before 5 years old may result in catching up to overall average levels in developmental trajectories, whereas the remainders may develop ID (Pickles et al., 2014). Furthermore, patients who have lower cognitive abilities are more likely to obtain an identifiable genetic risk variant than those with a higher IQ (Sanders et al., 2015). Interestingly, our results showed that in the subgroup of ASD children with DD, children with genetic variants had lower language competence. In other words, children with lower language competence had a greater chance of finding genetic variants. Nudel et al. (2021) considered it as pleiotropy between language impairment and ASD. They observed a significant genetic overlap between specific language impairment and childhood autism (which excluded Asperger’s syndrome). Another hypothesis is that children with genetic conditions are more likely to display delays in early developmental milestones, especially in language and motor functions. Compared with idiopathic ASD, children with *PPP2R5D, ADNP*, *ASXL3, DYRK1A, MED13L* variants and so on were marked by extensive delays, 2.7 times for single words and 5.7 times for combined words (Wickstrom et al., 2021). Thus, ASD children with DD or ID, especially those with lower language competence, are recommended for genetic testing.

In our subjects, 2 patients had *de novo AXSL3* variants. It is a transcriptional regulator that belongs to a group of vertebrate asx-like proteins. The *ASXL3* gene is highly expressed in the cerebral cortex as an epigenetic regulator that plays a role in regulating and controlling gene expression through chromatin remodeling (Katoh and Katoh, 2004; Katoh, 2015). Most *ASXL3* variants are *de novo*, placing it among the top 10 neurodevelopmental genes with the highest frequency of *de novo* variants (Wright et al., 2015). The characteristics of *ASXL3*-related syndrome (also called Bainbridge-Ropers syndrome) are DD/ID (moderate to severe), language impairment or absent speech, hypotonia and dysmorphic facial features. Our patients had typical phenotypic characteristics, such as feeding difficulties and delayed motor and language abilities. However, they had only mild developmental delay with IQ/DQ higher than 55, and no obvious signs of hypotonia or epilepsy compared with other patients with *ASXL3* variants (Katoh and Katoh, 2004; Katoh, 2015). Although most *ASXL3*-related syndromes rely on molecular confirmation, many individuals with pathogenic variants of *ASXL3* can be identified by a combination of clinical symptoms and unique phenotypes and do not omit those with mild developmental delays.

## Conclusion

Our work shows the utility of TSP, which has lower cost and more efficient genetic diagnosis and confirms the effectiveness of the test strategy. TSP should be offered to ASD patients in the expectation of preventative guidance and early detection of comorbidities. Subtypes of ASD children, especially those with language deficits, are recommended for testing to help families develop better intervention strategies.

## Data Availability

The data presented in the study are deposited in the GSA-Human repository, accession number HRA003901.
